# Carbonic anhydrase related protein expression in astrocytomas and oligodendroglial tumors

**DOI:** 10.1186/s12885-018-4493-4

**Published:** 2018-05-23

**Authors:** Sini L. Karjalainen, Hannu K. Haapasalo, Ashok Aspatwar, Harlan Barker, Seppo Parkkila, Joonas A. Haapasalo

**Affiliations:** 1 Faculty of Medicine and Life Sciences University of Tampere, Arvo Ylpön katu 34, 33014 Tampere, Finland; 2 Fimlab Laboratories, Department of Pathology, Tampere University Hospital, Biokatu 4, PL 2000, 33521 Tampere, Finland; 3 Unit of Neurosurgery, Tampere University Hospital, Teiskontie 35, 33521 Tampere, Finland

**Keywords:** CARPs, Glioma, Tumorigenesis, Astrocytomas, Oligodendroglioma, Immunohistochemistry

## Abstract

**Background:**

Carbonic anhydrase related proteins (CARPs) VIII, X and XI functionally differ from the other carbonic anhydrase (CA) enzymes. Structurally, they lack the zinc binding residues, which are important for enzyme activity of classical CAs.

The distribution pattern of the CARPs in fetal brain implies their role in brain development. In the adult brain, CARPs are mainly expressed in the neuron bodies but only weaker reactivity has been found in the astrocytes and oligodendrocytes. Altered expression patterns of CARPs VIII and XI have been linked to cancers outside the central nervous system.

There are no reports on CARPs in human astrocytomas or oligodendroglial tumors. We wanted to assess the expression of CARPs VIII and XI in these tumors and study their association to different clinicopathological features and tumor-associated CAs II, IX and XII.

**Methods:**

The tumor material for this study was obtained from surgical patients treated at the Tampere University Hospital in 1983–2009. CARP VIII staining was analyzed in 391 grade I-IV gliomas and CARP XI in 405 gliomas.

**Results:**

CARP VIII immunopositivity was observed in 13% of the astrocytomas and in 9% of the oligodendrogliomas. Positive CARP XI immunostaining was observed in 7% of the astrocytic and in 1% of the oligodendroglial tumor specimens. In our study, the most benign tumors, pilocytic astrocytomas, did not express CARPs at all. In WHO grade II-IV astrocytomas, CARPs were associated with molecular events related to more benign behavior, which was the case with CARP VIII in oligodendrogliomas and oligoastrocytomas as well.

**Conclusions:**

The study observations suggest that the CARPs play a role in tumorigenesis of diffusively infiltrating gliomas. Furthermore, the molecular mechanisms beneath the cancer promoting qualities of CARPs have not yet been discovered. Thus, more studies concerning role of CARPs in oncogenesis are needed.

**Electronic supplementary material:**

The online version of this article (10.1186/s12885-018-4493-4) contains supplementary material, which is available to authorized users.

## Background

Carbonic anhydrases (CAs) catalyze the reversible conversion of carbon dioxide and water to bicarbonate ion and proton. These enzymes are fundamental in normal physiological processes in organisms [1, 2]. The role of the CAs in diseases has been investigated extensively, and at least CA II, IX and XII have been associated to neoplastic growth [3–5].

Three inactive CA isoforms were discovered in 1990s and were named carbonic anhydrase related proteins (CARPs) VIII, X, and XI [1, 6]. They lack the one or more of the three zinc binding histidine residues which are required for the enzymatic activity of CAs [1]. The distribution pattern of CARPs in adult and fetal brain implies a role in brain development [7]. In the human enteric nervous system, CARP VIII and X are expressed in neural cell bodies, whereas CARP XI is absent [7]. In the human central nervous system (CNS), weak but significant CARP X mRNA expression has been detected in almost all parts of the system, whereas it is absent in the fetal brain and other developing tissues. In the fetal brain, CARP VIII and XI expression has been detected first at the 84th day of gestation in the neuroprogenitor cells. Later expression has been found in the migrating neural cells. CARP X expression has been observed in the cerebral cortical neurons at the 141st day of gestation [8].

Immunohistochemical analysis has shown strong expression of CARP VIII in neuron bodies of almost all parts of the adult brain. Weak reactivity was observed in the astrocytes, while the expression was completely absent in the oligodendrocytes. Choroid plexus and pia arachnoid have been strongly positive for CARP VIII. The CARP XI expression is weaker than CARP VIII, but the overall distribution among the neurons and glial cells is similar. CARP X expression differs from the other CARPs; it has been found mainly in the myelin sheath of the oligodendrocytes, and only weak expression has been detectable in the Purkinje cells and neurons of olivary nuclei [8].

CARP VIII is predominantly expressed in cerebellar Purkinje cells and appears to modulate IP3-induced calcium release in these cells [9, 10]. A homozygous missense mutation in human *CA8* gene that codes for CARP VIII, leads to reduction in cerebellar volume causing mild mental retardation and cerebellar ataxia and some patients exhibit quadrupedal gait [11, 12]. In waddles mice, *Car8* gene mutation and the consequential protein deficiency are associated with obtrusive gait disorder [13]. In zebrafish, knockdown of the *CA8* gene leads to reduction in cerebellar volume and abnormal swim pattern, which are similar to the consequences of *CA8* gene mutation in humans and mice [14]. Thus, CARP VIII plays an important role in motor control in humans, mice and zebrafish.

The function of CARP X and XI is unclear in humans. However, expression studies in humans show that CARP X is highly expressed in pineal gland at night suggesting its role in circadian rhythm (day-night cycle) [15]. The 5′-untranslated region of the *CA10* gene contains a CCG trinucleotide repeat in normal humans. The presence of CCG repeats in the *CA10* gene indicates that CARP X could be involved in neurologic disorders due to mutations expanding the number of repeats [16]. Studies in humans and cultured cells showed that CARP XI is associated with spinocerebellar ataxia 3/Machado Joseph’s disease [17]. The latest studies on CARP X and CARP XI in zebrafish showed that these genes are predominantly expressed in CNS, and knockout of these genes induces to apoptosis in the brain leading to ataxic swim pattern in the *ca10a* and *ca10b* mutant zebrafish [18].

Altered expression patterns of CARPs VIII and XI have been also linked to cancer. CARP VIII has been reported to promote colon cancer cell growth and invasiveness [19]. In colorectal adenocarcinomas, strong expression of CARP VIII has been found at the tumor infiltrative border [20]. In lung adenocarcinoma cells, CARP VIII increases cell growth in a laminin-rich environment, which led to a hypothesis that it may interrupt apoptosis signaling [21]. Strong CARP VIII expression has been observed in the developing fetal lung and at the infiltrative border of the non-small cell lung carcinoma, but only minor expression has been found in the adult lung [22]. Most of the gastrointestinal stromal tumors (GIST) overexpress both CARP VIII and XI, and especially CARP XI seems to enhance proliferation and invasion of these tumors [23]. Similar to the lung and colorectal cancers, the most intense expression of CARPs VIII and XI is present in the marginal areas rather than the center of the GISTs [23].

Recent study showed an increased expression of *CA8* gene in more aggressive types of human osteosarcoma (HOS) cells and the *CA8* expression correlated with the disease stages, showing intense expression in the late stages of the disease. Further experiments involving overexpression of *CA8* gene in HOS cells showed significant increase in cell proliferation both in vitro and in vivo. Interestingly, downregulation of *CA8* gene in HOS cells decreased cell invasion and colony formation ability in soft agar, and reduced the expression of metalloproteinase 9 (MMP9) and focal adhesion kinase (FAK), suggesting a role for CARP VIII in cancer cell invasion through the activation of FAK-MMP9 signaling [24]. Even though several tumors overexpress CARPs, the exact molecular mechanisms beneath their cancer promoting qualities have not yet been discovered [19, 23].

In the case of brain tumors, there have been no reports on CARPs in human astrocytomas and oligodendroglial tumors (oligodendrogliomas and oligoastrocytomas). These tumors derive from glial cells or glial progenitor cells and the majority of them are highly malignant. The surgical treatment is often inadequate and the prognosis of patients is still poor [25, 26]. Because the progenitor cells have been shown to express CARPs during fetal development, we wanted to assess the expression of different CARPs in astrocytomas, oligodendrogliomas and oligoastrocytomas, and to study their association with different clinicopathological features and patient survival. Since CAs II, IX and XII have shown high expression in gliomas, their association with CARPs was also evaluated.

## Methods

### Materials

The tumor material was obtained from surgical patients treated at the Tampere University Hospital in 1983–2004 (astrocytomas) and in 1983–2009 (oligodendroglial tumors). The study was approved by the research ethics committee of Tampere University Hospital. Brain tumor specimens fixed in 4% phosphate-buffered formaldehyde were processed into paraffin blocks. The tissue sections were stained with hematoxylin and eosin and then evaluated according to the WHO 2007 criteria [27] by a neuropathologist. A sample from a histologically representative tumor region was included in multi-tissue blocks constructed with a custom-built instrument (Beecher Instruments, Silver Spring, MD, USA). The tissue cores were 600 μm in diameter.

There were 327 diffusely infiltrating astrocytoma (WHO grades II-IV) samples of which 255 were primary tumors and 72 were recurrences. The ages of patients with primary tumors varied from 17 to 91 years (mean 64). In addition, the material included 31 grade I pilocytic astrocytomas (20 primaries of age 11–74 (mean 24) years, and 11 recurrences).

The oligodendroglial tumor (WHO grades II-III) group included 86 tumor samples, of which 69 were primary tumors and 17 were recurrences. The ages of patients with primary tumors varied from 5 to 74 (mean 49) years.

### Immunohistochemistry

Automated immunostaining for CARP VIII and XI was performed using BrightVision+ Poly-HRP-Anti IHC Kit (Poly-HRP-Anti-mouse/rabbit/rat IgG immunohistochemistry kit; ImmunoLogic, Duiven, The Netherlands) reagents. The immunostaining method included the following steps: (a) rinsing in wash buffer; (b) treatment in 3% hydrogen peroxide in double-distilled water (ddH_2_O) for 5 min and rinsing in wash buffer; (c) blocking with cow colostrum diluted 1:5 in dH_2_O for 10 min and rinsing in wash buffer; (d) incubation with rabbit polyclonal anti-CA VIII or anti-CA XI antibodies (Santa Cruz Biotechnology CA VIII: SC-67330 Lot#A2808, CA XI: SC-67333 Lot#A2808) both diluted 1:250 in Universal IHC Blocking/Diluent (ImmunoLogic Normal Antibody Diluent) for 30 min. The brain tumor specimens have also been immunostained with antibodies against CA IX (positive controls) and normal rabbit serum (negative controls) as previously described [28]; (e) rinsing in wash buffer for 3 × 5 min; (f) incubation in poly-HRP–conjugated anti-rabbit immunoglobulin G for 30 min and rinsing in wash buffer for 3 × 5 min; (g) incubation in 3,3′-diaminobenzidine tetrahydrochloride (Vector ImmPact DAB) solution (1 drop of ImmPACT™ DAB Chromogen concentrate to 1 ml ImmPACT™ DAB Diluent) for 5 min; (h) rinsing with water; (i) copper(II) sulfate treatment for 5 min to enhance the signal; (j) rinsing in wash buffer; (k) rinsing with 1/3 hematoxylin/ddH_2_O for one minute. All procedures were carried out at room temperature.

The intensity of cytoplasmic staining reactions was scored from the tissue microarrays on a scale from 0 to 3: 0, no reaction; 1 (+), weak reaction; 2 (++), moderate reaction; and 3 (+++), strong reaction. In the statistical analyses, the specimens were grouped into two large categories based on the staining: CARP-positive tumors with weak, moderate or strong immunostaining and CARP-negative tumors with negative immunostaining results. EGFR amplification was detected with EGFR chromogenic in situ hybridization (CISH) as described previously described [29]. In the analysis immunopositivity of VEGF, EGFR and CA II is marked as 1, negative immunostaining is marked as 0.

### Statistical analysis

All of the statistical analyses were performed using SPSS for Windows (SPSS 20.0, Chicago, IL, US). The significances of associations were defined using the chi-square test. The significance of associations was defined using χ2 test.

### FANTOM expression of CARP genes

The functional annotation of the mammalian genome (FANTOM) project has performed expression analysis on 1839 samples from 573 primary cells, 152 tissues, and 250 cell lines in human. Specifically, the data comes from cap analysis gene expression (CAGE) sequencing of cDNA [30]. We retrieved expression values for *CA8* and *CA11* from the FANTOM database (http://fantom.gsc.riken.jp/5/sstar/) in units of normalized tags per million (TPM).

### Computational analysis of transcription factor binding sites in CARP promoters

A computational comparative genomics analysis was performed on the promoter region of the human *CA8* (Ensembl: ENSG00000178538) and *CA11* (Ensembl: ENSG00000063180) genes, defined as the region 800 nucleotides (nt) upstream and 200 nt downstream of the transcription start site (TSS). There is currently just one *CA8* transcript identified as protein coding, ENST00000317995, which encodes the full-length 290 amino acid (aa) *CA8* protein. There are two *CA11* transcripts, yet only one, ENST00000084798, expresses the full-length 328aa CA11 protein. A second transcript, ENST00000596080, produces a truncated 113aa *CA11* protein. In our analysis we only addressed the *CA11* transcript corresponding to the full-length protein.

For both *CA8* and *CA11* transcripts, an alignment of the corresponding promoter nucleotide sequences of available mammalian species (including human) was retrieved from the Ensembl database, 17 species and 24 species, respectively. Using the Python tool tfbs_footprinter from the TFBS_footprinting package (https://pypi.python.org/pypi/TFBS-footprinting), experimentally determined binding sites for transcription factors (TFs) from the Jaspar database [31] were used to construct position weight matrices and subsequent log likelihood scoring of potential transcription factor binding sites (TFBSs). After scoring all possible positions in the nucleotide sequences of all species, locations where high scoring potential TFBSs were observed in multiple species were identified.

## Results and discussion

The cellular origin of the gliomas has been a focus of intensive research for decades. Many recent studies have implied that the neural stem cells can initiate and maintain the growth of human brain tumors, and hence could act as considerable targets for therapy [25]. In the development of CNS, fetal neuroprogenitor cells express CARP VIII and XI [8]. Mature oligodendrocytes, excluding the myelin sheath, do not express CARPs at all. Astrocytes have shown weak expression of CARPs VIII and XI [8]. The presence of CARPs in both the fetal brain and some tumors suggests that they might represent oncofetal proteins and play a role in the development of gliomas.

**Fig. 1:**
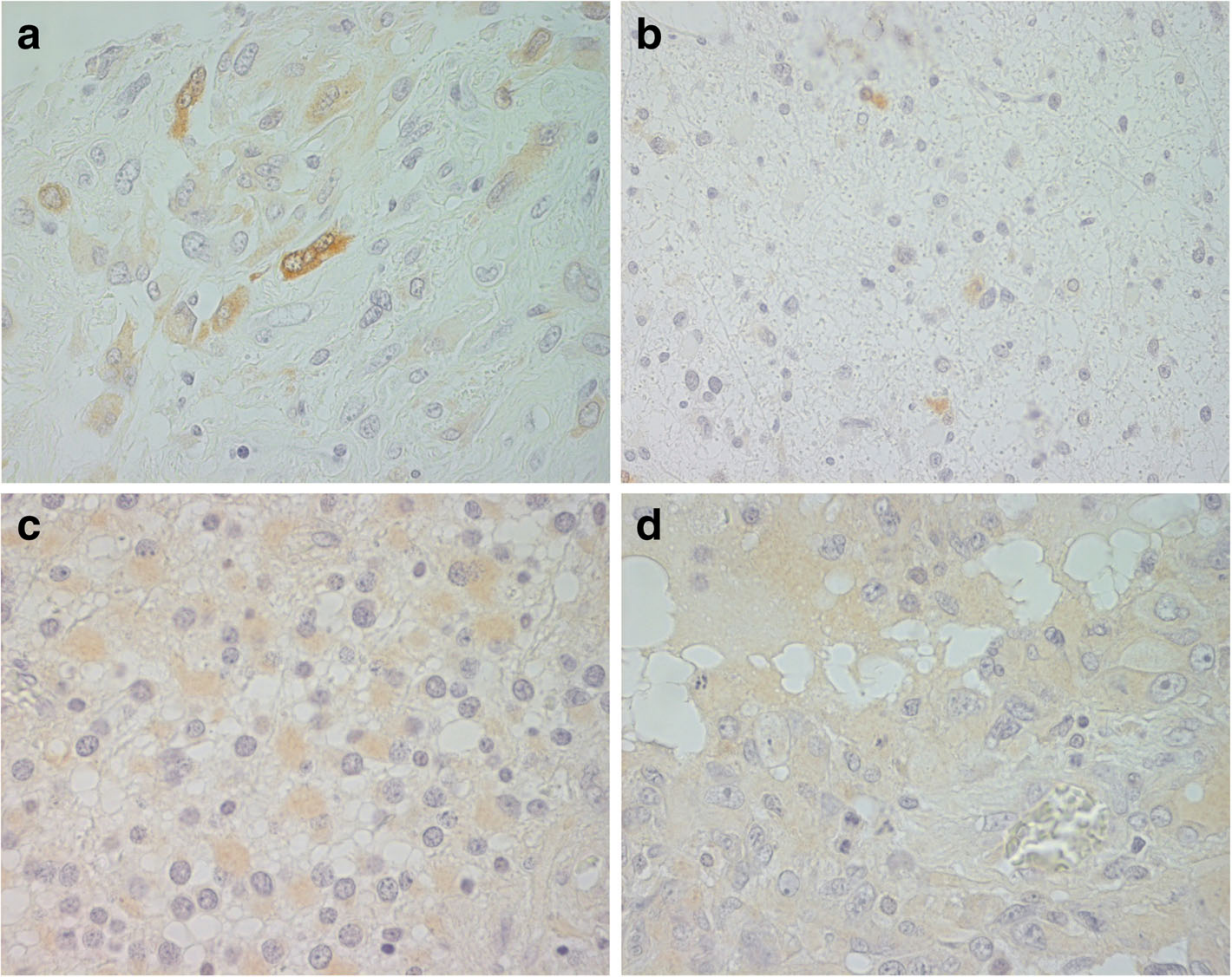
Immunohistochemistry. **a** CARP VIII in glioblastoma (gr IV). Strongly positive cytoplasmic immunostaining in two cells (magnification × 630). **b** CARP VIII in astrocytoma gr II. Moderately positive cytoplasmic staining focally (× 400). **c** CARP XI in astrocytoma gr III. Moderate cytoplasmic immunostaining in most of the cells (× 630). **d** CARP XI in glioblastoma (gr IV), Weak and diffuse cytoplasmic staining (× 630)

In this study, we first evaluated the expression of CARPs VIII, X and XI in astrocytomas, oligodendrogliomas and oligoastrocytomas by immunohistochemistry. Because the expression of CARP X turned out to be extremely low in gliomas, it was excluded from the final analyses. Some representative images of CARP VIII and XI staining are shown in Fig. 1.

CARP VIII staining was analyzed in **391** grade I-IV gliomas and CARP XI in **405** gliomas (Table 1). Of these no CARP immunostaining was observed in grade I pilocytic astrocytomas (excluded in the further analyses).

**Table 1:** CARP VIII and CARP XI expression in astrocytic and oligodendroglial gliomas (WHO 2016 CNS tumor classification)

Histological type	Histological grade	CARP VIII				CARP XI			
		Negative	Weak	Moderate	Strong	Negative	Weak	Moderate	Strong
Pilocytic astrocytoma	I	31	0	0	0	30	0	0	0
Diffuse astrocytoma, IDH-mutant	II	4	1	0	0	4	0	0	0
Gemistocytic astrocytoma, IDH-mutant	II	4	0	0	0	4	0	0	0
*Diffuse astrocytoma, IDH-wildtype*	II	3	0	0	0	3	0	0	0
Diffuse astrocytoma, NOS	II	24	2	7	0	30	3	0	0
Anaplastic astrocytoma, IDH-mutant	III	5	0	0	0	5	0	0	0
*Anaplastic astrocytoma, IDH-wildtype*	III	1	0	0	0	1	0	0	0
Anaplastic astrocytoma, NOS	III	33	2	2	0	30	4	1	0
Glioblastoma, IDH-wildtype	IV	163	8	8	4	163	12	0	0
Giant cell glioblastoma	IV	3	0	1	0	4	0	0	0
Gliosarcoma	IV	12	0	1	0	11	1	0	0
Glioblastoma, IDH-mutant	IV	16	2	3	0	19	1	0	0
Glioblastoma, NOS	IV	14	2	1	1	16	1	0	0
Oligodendroglioma, IDH-mutant and 1p/19q-codeleted	II	21	2	1	0	23	0	0	0
Oligodendroglioma, NOS	II	11	2	1	0	15	0	0	0
Anaplastic oligodendroglioma, IDH-mutant and 1p/19q-codeleted	III	10	0	0	0	10	0	0	0
*Anaplastic oligodendroglioma, NOS*	III	8	0	0	0	8	0	0	0
Oligoastrocytoma, NOS	II	10	0	0	0	11	0	0	0
*Anaplastic oligoastrocytoma, NOS*	III	18	1	1	0	18	0	1	0

In further statistical analysis the grade II – IV gliomas with inconclusive genetic testing (according WHO 2016 CNS tumor classification) were also excluded. The excluded tumors are marked as NOS (not otherwise specified) tumors in the Table 1.

In diffuse astrocytic and oligodendroglial tumors tested for the appropriate molecular markers CARP XI expression differed significantly between astrocytomas and oligodendrogliomas; all oligodendrogliomas were negative (*p* = 0.040, chi-square test). In astrocytomas and oligodendrogliomas, there were no association between CARPs and proliferation by Ki-67 / MIB-1 (p = n.s, Kruskal-Wallis test).

In diffuse gliomas tested for the appropriate molecular markers CARP XI expression differed significantly between diffuse astrocytomas and oligodendrogliomas; all oligodendrogliomas were negative (p = 0.040, chi-square test). In diffusively infiltrating astrocytomas and oligodendrogliomas, there were no association between CARPs and proliferation by Ki-67 / MIB-1 (p = n.s, Kruskal-Wallis test).

In gliomas, hypoxia plays a key role in the regulation of tumor aggressiveness and treatment resistance. Hypoxia-inducible factors (HIF1 and 2) enhance tumor growth under hypoxic conditions by turning on several hypoxia-responsive genes, such as vascular endothelial growth factor (VEGF), epidermal growth factor receptor (EGFR) and, importantly, *CA9* which has been shown to be strongly controlled by hypoxia also in brain tumors [32]. Both EGFR and VEGF gene amplifications are usually associated to higher tumor grade and thus poorer outcome of patients. In gliomas, VEGF is mainly found in perinecrotic tumor areas. EGFR gene is amplified in 40% of the glioblastomas [33].

In our study, the majority of the tumors expressing either CARP VIII or CARP XI were negative concerning VEGF. Almost all CARP VIII-positive astrocytomas inversely correlated to VEGF (*p* = 0.005, chi-square test, *n* = 197. These observations suggest that the CARPs are not related to hypoxic conditions.

In gastrointestinal, colorectal and lung cancers, the most intense expression of CARPs has been reported in the tumor infiltrative border rather than in the hypoxic center areas of the tumors [20, 22, 23]. These findings imply that CARPs might play a specific role in tumor invasion. In our study, we were not able to define the precise intratumoral location of CARPs, because each tissue microarray specimen contained only a single representative area of the tumor. On the other hand, diffusely infiltrating gliomas usually invade either individually or in small groups of cells [34]. Obviously, analyses of larger sections or multiple intratumor biopsies are warranted to evaluate further CARP expression separately in hypoxic and invasive areas. Similarly, the role of tumor cell invasion should be tested in patient derived in vitro analysis in the future studies. In light of our increasing understanding of genetic diversity with sub-clones of malignant tumors and stem cells, CAs II, VII, IX and XII have been shown to be present in several categories of brain tumors [28, 31, 35–37]. CA IX seems to be the most interesting CA in terms of its potential role in brain tumor diagnostics and drug therapy [35]. Its transcription is facilitated by HIF-1 alpha [32]. In a recent study, a negative correlation was found between CA IX and enzyme cytosolic isocitrate dehydrogenase 1 (IDH1) mutation in astrocytomas [38]. In our study, no correlation was found between CARP VIII expression and the other CAs (p = n.s., chi-square test) or IDH1 mutations (p = n.s., chi-square test). Similarly, there was no correlation between CARP XI and IDH1, nor did CARP XI correlate with the expression of CA VII, CA IX, and CA XII (p = n.s., chi-square test). Interestingly, the expression of CARP XI positively correlated with the cytoplasmic CA II in astrocytic tumors (*p* = 0.037, chi-square test). Endothelial expression of CA II has been associated with neoplastic growth and considered a potential molecule in diagnostics and treatment of gliomas [39]. CA II is regulated mainly by factors other than hypoxia [40], which might be the case with CARPs as well. When primary and recurrent tumors were analyzed in different tumor types, no differences in CARP VIII and XI was found (p = n.s., chi-square test). 1p/19q co-deletion is an independent prognostic and predictive marker in oligodendroglial tumors [40]. In our study, all oligodendrogliomas (with defined 1p/19q – co-deletion) were CARP XI negative.

From the FANTOM project, the samples where expression of the CARP VIII CAGE peak is found most strongly are: small-cell gastrointestinal carcinoma, acute myeloid leukemia, cerebellum, rectal cancer, and reticulocytes; whereas the expression was extremely low in astrocytoma, glioblastoma, and glioma samples (Additional file 1: Table S1). Similarly, the top five samples where expression of the *CA11* CAGE peak is found most strongly are: middle temporal gyrus, medial temporal gyrus, amygdala, parietal lobe, and caudate nucleus; while also near zero in astrocytoma, glioblastoma, and glioma (Additional file 2: Table S2). In our study, CARP XI protein immunopositivity was observed only in one case (1%) of the group of oligodendrogliomas.

It is of interest that CARP VIII and XI are only weakly expressed in the mature astrocytes and they are absent in the oligodendrocytes [8]. In our study, the overall expression of the CARPs in the astrocytomas and oligodendroglial tumors. Was rather weak. The most benign tumors, pilocytic astrocytomas, did not express CARPs at all. When the grade II-IV astrocytomas and oligodendrogliomas were evaluated separately, no correlation to WHO tumor grade was found (p = n.s, chi-square test).

However, in a recent study the inhibition of *CA8* gene in human osteosarcoma cells sensitized the tumor cells to cisplatin chemotherapy [24]. Since this chemotherapy is also used in glioma treatment, it would be of interest to analyze the effect of *CA8* inhibition combined to cisplatin in gliomas.

**Fig. 2:**
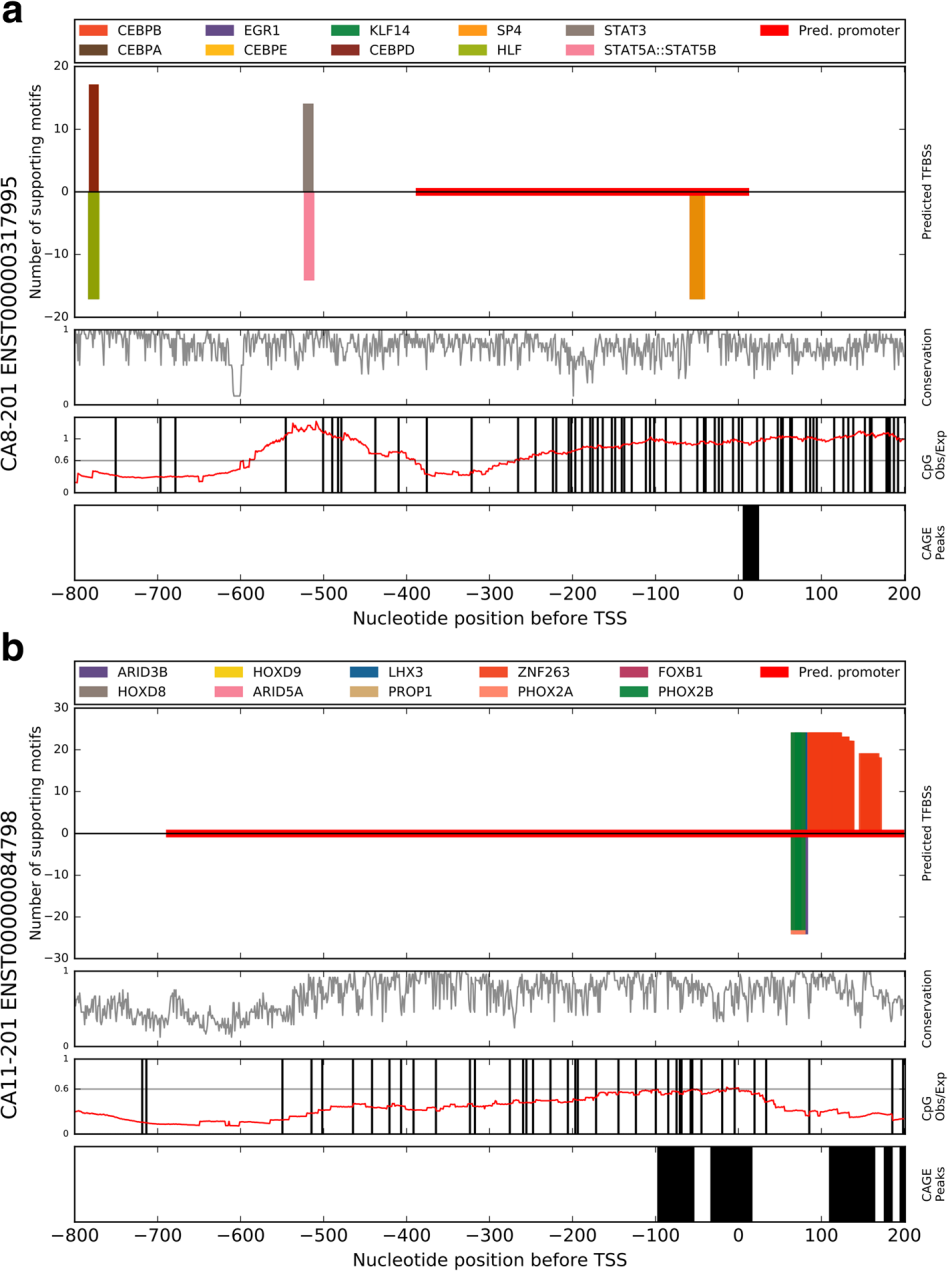
Comparative genomics TFBS analysis of the *CA8* and *CA11* promoters. Alignments of 17 and 24 mammal sequences corresponding to the promoters of full-length human *CA8* transcript ENST00000317995 (**a**) and *CA11* transcript ENST00000084798 (**b**), respectively, were analyzed for putative transcription factor binding sites by comparative genomics. For each, the 10 best scoring transcription factors were included in the figure, where height indicates the number of species supporting that prediction. Positive y-axis results indicate a TFBS predicted on the positive strand while negative y-axis indicates the negative strand. Conservation of sequence in the alignment, CpG ratio in the human sequence, and location of FANTOM CAGE peaks in the human sequence are included as subpanels. Location relative to TSS is marked across the bottom of the figure, and is relevant for all panels

Our analysis of the *CA8* promoter has identified a number of putative TFBSs which may affect *CA8* expression (Fig. 2). CAGE data from the FANTOM project shows a peak near the Ensembl defined TSS. This peak overlaps, and is supported by, an Ensembl predicted promoter region. Within this promoter region our analysis has predicted a cluster of overlapping TFBSs within an area that is − 59 to − 41 nt upstream of the TSS. Specifically, the TF proteins, which are predicted to bind here, are KLF14, SP4, and EGR1. Two additional binding clusters exist further upstream of the TSS, STAT5A:STAT5B and STAT3 at − 524 to − 512, and a site for CEBPA, HLF, CEBPE, CEBPD, and CEBPB at − 783 to − 771. A complete list of predictions is available as Additional file 3: Table S3.

Early growth response-1 (EGR1) has demonstrated both tumor-suppressor and oncogene properties depending on the type of tumor. In high-grade astrocytoma, EGR1 expression is associated with enhanced patient survival [41]. However, Sakakini et al. recently observed that EGR1 expression was much more consistently observed in glioblastoma than in pilocytic astrocytomas, that in proliferating cyclin A/OLIG2-positive cells EGR1 was localized to the nucleus, and that this produced increased aggressiveness and stemness [42]. Additional links are found between glioma and STAT3 and CEBPB TFs. Specifically, these two proteins correlate with mesenchymal differentiation and poor clinical outcome [43]. STAT5B inhibition reduces glioblastoma cell growth, cell cycle progression, invasion, and migration [44]. There are a number of candidate TFs, across three clusters of binding sites, that have established roles in astrocytoma and glioblastoma. Based on expression values from the extensive FANTOM project, we see that *CA8* is expressed highly in cerebellum (95.76 TPM) but near zero in astrocytoma, glioblastoma, and glioma. With the significantly higher *CA8* expression in normal brain tissues than in tumor tissues, these putative TFBSs would make interesting subjects for future study as possible repressors of *CA8*.

Our analysis of the *CA11* promoter showed that the strongest predicted binding sites are in two distinct clusters (Fig. 2). The first cluster is comprised entirely of numerous ZNF263 binding sites on the positive strand overlapping the most highly expressed FANTOM CAGE peak, locations + 86 to + 132 nt relative to TSS. The second cluster of predicted TFBSs all overlap one another immediately upstream of the same most strongly expressed CAGE peak, and on both positive and negative DNA strands: LHX3, ARID3B, PHOX2A, PROP1, PHOX2B, FOXB1, ARID5A, HOXD8, and HOXD9 at locations + 64 to + 83 nt. A complete list of predictions is available as Additional file 4: Table S4.

Compared to the *CA8* promoter analysis, these predictions for potential TFBSs were more limited in their correlation with astrocytoma. However, this makes sense as nearly all the best scoring predictions seemed to occupy one position. PHOX2B has been identified to have a hypermethylated promoter in glioma, thus resulting in its down-regulation [45]. Similarly, a CpG locus in the HOXD8 gene 5’ UTR is differentially hypermethylated in short-term survival gliomas vs. long-term survivors [46]. Increased expression of HOXD9, the other HOX family member predicted at this location, has been found associated with cell proliferation, and may be a marker of glioma stem cells [47]. The prolific set of potential ZNF263 binding sites overlapping the CAGE peak could be a locus for *CA11* repression, as ZNF263 serves that function in some genes [48]. These two proximal sites represent potential regulatory sites worth investigating further to understand the decreased expression of *CA11* in astrocytoma and oligodendroglioma.

## Conclusions

Our studies showed no CARP expression in pilocytic astrocytomas. In higher grade gliomas, the CARP expression is mainly absent or weak. In this study, the CARP VIII and XI proteins were associated to non-hypoxic conditions and CARP XI also to the expression of cytoplasmic CA II staining. The expression of CARPs did not associate to survival or tumor WHO grade. Due to their presence in a subset of malignant gliomas, CARPs may have a role in tumorigenic processes. However, further investigations on the functions of CARPs are warranted.

## Additional files

Additional file 1: **Table S1.** Expression of *CA8* CAGE peak, as derived from the FANTOM project. (XLSX 38 kb)

Additional file 2: **Table S2.** Expression of *CA11* CAGE peak, as derived from the FANTOM project. (XLSX 45 kb)

Additional file 3: **Table S3.** Full results of comparative genomics prediction of TFBSs in *CA8* promoter. (XLSX 82 kb)

Additional file 4: **Table S4.** Full results of comparative genomics prediction of TFBSs in *CA11* promoter. (XLSX 105 kb)
